# P-1534. Development of PCRSeqTyping to Differentiate Streptococcus pneumoniae (SP) Serotypes 15B and 15C

**DOI:** 10.1093/ofid/ofaf695.1715

**Published:** 2026-01-11

**Authors:** Ilan Rozen Eisenberg, Yazdani Shaik-Dasthagirisaheb, Stephen Pelton

**Affiliations:** Boston Medical Center / Boston University, Brookline, MA; Boston Medical Center / Boston University, Brookline, MA; Boston Medical Center / Boston University, Brookline, MA

## Abstract

**Background:**

*S. pneumoniae* (SP) serotypes 15B and 15C combined are amongst the most frequent causative agents of invasive pneumococcal disease and nasopharyngeal colonization in the post PCV13 era. The introduction of PCV20 included serotype 15B polysaccharide conjugates in its formulation, but not 15C. It remains unclear whether including 15B polysaccharide with its dominant O-acetyl epitope will elicit protection against both serotypes.

Current practices to differentiate between serotypes relay on conventional methods and serotype determination with specific antisera, limiting our ability to evaluate serotype in the culture negative samples. Current molecular serotyping strategy, published by CDC, identifies up to 20 vaccine serotypes, including serotype 15B, but do not differentiate 15B from 15C. The principal difference between serotypes 15B and 15C is the presence of an *O*-acetyl group on the pentasaccharide-repeating unit of 15B polysaccharide. The O-acetylation is controlled by the number of AT pairs; with 8 pairs turning on O-acetylation (15B) and 7 or 9 turning off acetylation (15C). An accurate and rapid serotyping method is essential to differentiate serotypes 15B and 15C, and evaluate whether PCV20 will provide protection against both serotypes.

**Figure:**
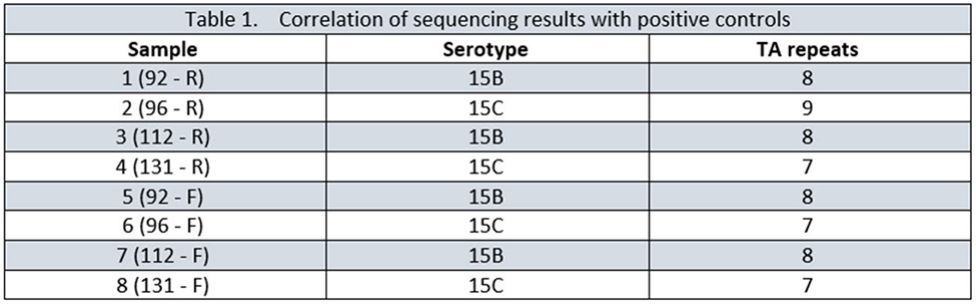

**Methods:**

DNA extraction of SP negative samples were performed. We isolated DNA from known strains of 15B and 15C to serve as positive controls. LytA and PiaB genes were used to confirm SP by QRT-PCR, followed by molecular serotyping using serotype specific primers and probes. Samples positive for 15 BC were further amplified by PCR using O-Acetyl Transferase gene specific primers. PCR DNA was purified and submitted for the sequencing with O-acetyl transferase specific primers.

**Results:**

A 100% correlation of PCRSeqTyping results was observed using sequencing results with positive controls. Eight culture negative samples were identified correctly as 15B (n=4) or 15C (n=4

**Conclusion:**

We describe a PCRSeqTyping assay to differentiate 15B from 15C that is accurate and rapid, with high reproducibility. It is significant as it can allow differentiation of 15B and 15C on culture negative specimens. This assay will enhance surveillance studies and enable researchers to better determine if PCV20 provides protection against both 15B and 15C.

**Disclosures:**

All Authors: No reported disclosures

